# Single nucleotide polymorphisms in candidate genes associated with milk yield in Argentinean Holstein and Holstein x Jersey cows

**DOI:** 10.1186/s40781-018-0189-1

**Published:** 2018-12-12

**Authors:** María Agustina Raschia, Juan Pablo Nani, Daniel Omar Maizon, María José Beribe, Ariel Fernando Amadio, Mario Andrés Poli

**Affiliations:** 1 Instituto Nacional de Tecnología Agropecuaria (INTA), Centro de Investigación en Ciencias Veterinarias y Agronómicas (CICVyA), Instituto de Genética “Ewald A. Favret”, Nicolás Repetto y de los Reseros s/n, Hurlingham, B1686 Argentina; 2 Instituto Nacional de Tecnología Agropecuaria (INTA), Estación Experimental Agropecuaria Rafaela, Ruta Nacional 34 Km 227, Rafaela, Argentina; 3 Instituto Nacional de Tecnología Agropecuaria (INTA), Estación Experimental Agropecuaria Anguil, Ruta Nacional 5 Km 580, Anguil, Argentina; 4 Instituto Nacional de Tecnología Agropecuaria (INTA), Estación Experimental Agropecuaria Pergamino, Ruta 32 Km 4.5, Pergamino, Argentina; 5 Consejo Nacional de Investigaciones Científicas y Técnicas (CONICET), Ciudad Autónoma de Buenos Aires, Argentina

**Keywords:** Milk production, Dairy cattle, Candidate genes, Association study, Single nucleotide polymorphisms

## Abstract

**Background:**

Research on loci influencing milk production traits of dairy cattle is one of the main topics of investigation in livestock. Many genomic regions and polymorphisms associated with dairy production have been reported worldwide. In this context, the purpose of this study was to identify candidate loci associated with milk yield in Argentinean dairy cattle. A database of candidate genes and single nucleotide polymorphisms (SNPs) for milk production and composition was developed. Thirty-nine SNPs belonging to 22 candidate genes were genotyped on 1643 animals (Holstein and Holstein x Jersey). The genotypes obtained were subjected to association studies considering the whole population and discriminating the population by Holstein breed percentage. Phenotypic data consisted of milk production values recorded during the first lactation of 1156 Holstein and 462 Holstein x Jersey cows from 18 dairy farms located in the central dairy area of Argentina. From these records, 305-day cumulative milk production values were predicted.

**Results:**

Eight SNPs (rs43375517, rs29004488, rs132812135, rs137651874, rs109191047, rs135164815, rs43706485, and rs41255693), located on six *Bos taurus* autosomes (BTA4, BTA6, BTA19, BTA20, BTA22, and BTA26), showed suggestive associations with 305-day cumulative milk production (under Benjamini-Hochberg procedure with a false discovery rate of 0.1). Two of those SNPs (rs43375517 and rs135164815) were significantly associated with milk production (Bonferroni adjusted *p*-values < 0.05) when considering the Holstein population.

**Conclusions:**

The results obtained are consistent with previously reported associations in other Holstein populations. Furthermore, the SNPs found to influence bovine milk production in this study may be used as possible candidate SNPs for marker-assisted selection programs in Argentinean dairy cattle.

**Electronic supplementary material:**

The online version of this article (10.1186/s40781-018-0189-1) contains supplementary material, which is available to authorized users.

## Background

Within Argentina’s economy, the dairy industry is considered one of the most important and dynamic agrifood sector. Its structure is the result of a process of concentration and specialization of several years, with a decrease in the number of dairy farms and an increase in their productive scale [1]. Currently, this country has approximately 1.8 million dairy cows distributed in 11,750 productive units [2]. The average milk production for the period 2011–2015 was of 11,167.9 million of liters/year [3]. From the total milk production, approximately 7.5% is sold through the informal market and/or consumed by farm households, while 92.5% is processed as fluid milk or as manufactured dairy products [4]. As production capacity far exceeds the volume required to meet domestic demand, between 15 and 25% of the total milk production is sold to external markets [3]. Dairy production system is primarily based on extensive grazing; however, an increased use of supplements is giving rise to a mixed system. Regarding current breeding strategies, there is no national scheme with a single objective but each producer follows its own strategy. Furthermore, at present, no breeding strategies using genomic information are being applied at national level.

Most applications of genetic information in selection programs are preceded by analyses aimed at quantitative trait loci (QTL) detection, which should be conducted in the populations that are going to be used for genetic improvement [5, 6]. Only QTLs that are shown to have a significant effect on phenotype are subsequently used for selection [5]. In this context, extensive genetic research on lactation and milk production in cattle has been performed. Consequently, several countries implemented in their breeding programs or in their genetic evaluation systems the information made available on many candidate regions, QTLs, and genome-wide molecular markers, for genetic improvement on milk production traits. France [7], Germany [8] and New Zealand [9] applied marker-assisted selection programs in dairy cattle breeding. Furthermore, genomic selection strategies were implemented in the United States, Canada, Great Britain, Ireland, New Zealand, Australia, France, the Netherlands, Germany, and the Scandinavian countries [10].

The criteria for selecting candidate regions, genes, and markers combine information from different sources that support the candidate status of these regions. Key genes responsible for milk production or composition traits should be identified on the basis of a multidisciplinary approach combining different pieces of evidence such as gene location, gene expression profile, regulation of gene expression, function of coded protein, and previously reported associations between markers on the gene and the phenotypes under study. SNPs within genes directly, indirectly, or potentially related to those traits are convenient markers to use for the identification of causal loci.

Over the last decades, the advances in DNA technology have given rise to many high throughput SNP genotyping platforms, leading to automation and a significant reduction in costs. These advantages, along with the abundance and distribution of SNPs in the genome, facilitate studies on the statistical genetic association of neighboring alleles or linkage disequilibrium, with the objective to identify the location of genes influencing the variation in certain traits. Furthermore, SNPs are also important intrinsic candidates as causal variants of these traits [11–13].

To date, no studies in Argentinean Holstein and Holstein x Jersey cows have reported associations between genetic markers and milk production. The objective of this study was to detect associations between SNP markers in candidate genes suspected to influence milk production and composition traits, and 305-day cumulative milk production, predicted for a representative population of Argentinean dairy cattle. The information obtained aims to improve milk production breeding programs.

## Methods

### Animals, blood samples, and phenotypic records

The animals used belonged to 18 commercial dairy farms owned by a single dairy company located in the central dairy area of Argentina. Blood samples were taken from 1618 cows comprising 1156 Holstein and 462 Holstein x Jersey crosses, and semen samples were obtained from 25 bulls (20 Holstein and 5 Jersey).

The phenotypic records consisted of data from the official dairy control obtained during the first lactation of the cows under study. All the animals were kept in similar feeding and sanitary conditions. The cows were machine milked twice a day and milk records were taken every ~ 40 days. Every cow included in this study had at least four dairy records during their first lactation. Procedures for blood samples collection and for milk production evaluation were approved by the Institutional Committee for Care and Use of Experimental Animals (CICUAE) of the National Institute of Agricultural Technology (INTA) under protocol number 35/2010 and were carried out in strict accordance of the guidelines specified in the institutional manual.

### DNA extraction

Fresh blood was obtained from the jugular vein of cows using EDTA as anticoagulant. Genomic DNA was extracted from blood samples using a commercial kit (AxyPrep Blood Genomic DNA Miniprep Kit, Axygen Biosciences, Union City, CA, USA), following the manufacturer’s protocol. Genomic DNA from sires was obtained from semen straws using a standard phenol-chloroform extraction. DNA quantity and quality were assessed by measuring DNA absorbance at 260 nm and evaluating 260/280 and 260/230 absorbance ratios, respectively, using a NanoDrop™ 1000 spectrophotometer (Thermo Scientific, Wilmington, DE, USA).

### Gene and marker selection

The candidate genes were selected by combining different sources of information available on databases and public literature. The chosen genes were those that met at least one of the following criteria, proposed by Ogorevc et al. [14]: 1) the gene encodes a protein directly associated with milk components or milk metabolism that exists in different genetic variants; 2) markers on the gene have proven association with milk traits; 3) the gene expression profile is related to the phenotype under study; 4) the gene is located within a cattle QTL for the studied phenotype; 5) miRNAs directed to the mRNA of the gene are expressed in bovine mammary gland and regulate its expression post-transcriptionally.

Three strategies were employed for marker selection: 1) markers from candidate genes previously found to be associated with milk production or composition traits; 2) markers from genes not directly related to milk production, but significantly associated with the phenotype under study; 3) newly identified SNPs by sequencing candidate genes on the evaluated sire population.

Primers were designed to amplify regions in 14 candidate genes: *LEP, ABCG2, OPN, PPARGC1A, CSN1S1, CSN2, CSN3, LGB, DGAT1, GH, GHR, PRLR, LTF,* and *PRL*, using BatchPrimer3 [15]. The amplified region was sequenced both to check the existence of known polymorphisms and to discover new ones. Additional file 1 indicates primer sequences, GenBank accession numbers, length of the amplicons, and optimal annealing temperatures used.

The PCR reactions were performed using the enzyme Paq5000® DNA Polymerase and its reaction buffer (Agilent Technologies, Stratagene, Santa Clara, CA, USA) following the manufacturer’s instructions. Conditions for amplification were 94 °C for 3 min, followed by 35 cycles of 20 s at 94 °C, 20 s at an optimal annealing temperature, and 1 min at 72 °C. Reactions were ended with a 72 °C, 5 min final extension stage. Amplified fragments from sire DNA were visualized in agarose gels stained with ethidium bromide and then sequenced using BigDye® chemistry (Perkin-Elmer, Wellesley, MA, USA) according to the manufacturer’s protocol, on an ABI3130xl sequencer (Applied Biosystems, Foster City, CA, USA). Sequencing reactions were conducted with the same primers as those used for PCR reactions, with a 1:10 dilution of the PCR product as template. Sequence traces assembly was performed with Gap4 [16]. The positions of the SNPs in the chromosomes were determined by mapping the SNPs including their 200 bp context against assembly UMD3.1 [17] using the Exonerate software [18].

### Genotyping and quality control

Forty-six markers from 23 candidate genes were taken into consideration for the SNPlex genotyping panel design. Considered candidate genes comprised *ARL4A* (ADP ribosylation factor like GTPase 4A), *ETV1* (ETS variant 1), *SNX13* (sorting nexin 13), *LEP* (leptin), *LALBA* (lactalbumin alpha), *OLR1* (oxidized low density lipoprotein receptor 1), *ABCG2* (ATP binding cassette subfamily G member 2), *OPN* (osteopontin), *PPARGC1A* (peroxisome proliferator-activated receptor gamma coactivator 1-alpha), *CSN1S1* (casein alpha s1), *CSN2* (casein beta), *CSN3* (casein kappa), *LGB* (beta-lactoglobulin), *DGAT1* (diacylglycerol O-acyltransferase 1), *STAT5A* (signal transducer and activator of transcription 5A), *GH* (growth hormone), *FASN* (fatty acid synthase), *GHR* (growth hormone receptor), *PRLR* (prolactin receptor), *UTMP* (uterine milk protein precursor), *LTF* (lactotransferrin), *PRL* (prolactin), and *SCD1* (stearoyl-CoA desaturase). Selection criteria required nucleotide substitutions in those genes to involve only two possible bases and to map to only one position in the genome. Forty-six SNPs were proposed for custom assay design. However, seven of the selected markers were not included in the panel because they failed to meet the manufacturer’s specifications. As a result, the designed panel consisted of 39 markers from 22 candidate genes to be genotyped with the SNPlex Genotyping System platform (Applied Biosystems, Foster City, CA, USA; [19]) on the commercial population of Holstein and Holstein x Jersey cattle under study. Genotyping reactions and analyses were done following provider protocols using an ABI3130xl sequencer and GeneMapper v4.0 software (Applied Biosystems).

Call rate, as well as genotypic and allelic frequencies, were calculated for all genotyped SNPs. Genotyping quality assurance was performed using PLINK v1.07 [20]. Only the SNPs that satisfied the following criteria were retained: (a) minor allele frequency (MAF) > 5%, and (b) percentage of missing genotypes across all samples < 10%. After quality pruning, 22 SNPs were included in the analysis. Concerning animals, those with more than 10% missing genotypes across all SNPs were removed. Mendelian errors, accounting for all assigned genotypes that generate genotypic inconsistencies in sire-mother-daughter or sire-daughter familiar nucleus, were detected with PLINK and set to missing genotypes. As expected, loci in this population did not match Hardy-Weinberg postulates. Furthermore, SNPs associated with milk production might have been under selection pressure in the dairy population used and therefore they might not have been in Hardy-Weinberg equilibrium (HWE). For the reasons previously stated, no consideration was given to deviation from HWE in the quality control test.

### Prediction of 305-day cumulative milk production

Lactation curves were adjusted on the basis of days in milk production, through Legendre orthogonal polynomials of fourth degree [21]. All curves were treated as random regression models, which made it possible to adjust a lactation curve for each individual -random regression-, expressed as the deviation from an average population curve -steady regression- [22]. The 305-day cumulative milk production was predicted for 1618 cows with at least four production records during their first lactation (MJ Beribe, personal communication).

### Statistical analysis and association test

Associations between each individual SNP and the 305-day cumulative milk production of 1346 cows (those that passed the genotype quality control criteria) were calculated using a general linear model in PLINK by the following equation:

$$ {Y}_{ijklmn}=\mu ++{b}_i+{yb}_j+{herd}_k+{SYFL}_l+{sire}_m+{SNP}_n+{e}_{ijklmn} $$

where *y*_*ijklmn*_ is the 305-day cumulative milk production previously predicted, μ is the overall population mean, *b*_*i*_ is the breed fixed effect, *yb*_*j*_ is the year of birth fixed effect, *herd*_*k*_ is the farm fixed effect, *SYFL*_*l*_ is the combined fixed effect of season and year of first lactation, sire_m_ is the sire effect, *SNP*_*n*_ is the effect of the SNP genotype, and *e*_*ijklmn*_ is the random residual. The breed effect comprised five categories: 100, 75, 50, 25 and 12.5% of Holstein genetic background. Cows with production records were born between the years 2000 and 2006, belonged to 18 different herds, and were sired by 20 Holstein and 4 Jersey bulls. The fixed effect accounting for the combination of season and year of first lactation considered four seasons (fall, winter, spring, and summer) and five years (2004 to 2008). Association tests were conducted on the whole population (1346 cows) as well as on pure Holstein (978 animals) and on Holstein x Jersey crosses (368 cows) separately. The contribution of each covariate included in the model was checked by PLINK software for each association study performed, and only those found to have significant effects were considered.

To account for the risk of false positives due to the multiple testing problem, *p*-values were adjusted by Bonferroni correction and Benjamini-Hochberg procedure using PLINK. Bonferroni adjusted *p*-values < 0.05 were accepted to represent a proof of significant association, while Benjamini-Hochberg adjusted *p*-values < 0.1 were considered as indicators of suggestive associations.

Student’s *t*-tests were conducted to compare the 305-day cumulative milk production of cows with different genotypes, on each SNP detected as significantly associated with the trait under study.

## Results

### Gene and marker selection and SNP panel design

Twenty-two candidate genes from bovine loci involved in milk production and composition traits were retrieved from literature [11–13, 23–41] and different open access databases (PubMed, Cattle QTL Database, and Ensembl). The collected data included loci on 11 chromosomes (BTAs 4, 5, 6, 11, 14, 19, 20, 21, 22, 23 and 26), with the highest number of candidate genes on BTA6. The final list of 39 SNPs included in the SNPlex panel is indicated in Table 1.

**Table 1:** SNPs included in the SNPlex genotyping panel

Candidate gene	Marker	Location (BTA:bp)	Call rate	MAF	HWE *p*-value
*ARL4A*	**rs43375517**	4:20296999	98.1	0.39	0.14
*ETV1*	**rs42213673**	4:22111819	98.8	0.35	0.28
*SNX13*	**rs41595314**	4:26425057	98.6	0.07	8.10^− 3^
*LEP*	**rs29004488**	4:93262056	97.4	0.33	0.02
*OLR1*	rs109019599	5:100254823	89.3	0.25	2.10^−3^
*ABCG2*	rs43702337	6:38027010	99.7	0.00	1.00
*OPN*	**rs132812135**	6:38120968	94.5	0.35	0.55
	**rs110930453**	6:38122665	93.1	0.42	0.87
	OPN3907^a^	6:38128806			
*PPARGC1A*	**rs109579682**	6:44875251	98.4	0.23	0.03
	**rs133669403**	6:44875315	97.5	0.08	0.09
	**rs17870811**	6:44875421	99.5	0.06	0.46
*CSN1S1*	CSN1_AB	6:87146017	87.9	0.00	1.00
	rs433385179	6:87148464	99.9	0.00	1.00
	**rs43703010**	6:87157262	96.8	0.15	0.64
*CSN2*	rs43703013	6:87181453	96.8	0.04	0.02
	**rs43703011**	6:87181619	97.6	0.27	0.01
*CSN3*	rs43706475	6:87390479	99.3	0.00	1.00
	**rs43703015**	6:87390576	99.4	0.19	0.33
	rs43703017	6:87390632	98.2	0.04	0.33
*LGB*	rs41255680	11:103301242			
	rs41255685	11:103301690	75.3	0.08	< 1.10^−6^
	rs43691049	11:103303343			
	rs109625649	11:103304757	83.5	0.10	0.56
*DGAT1*	rs109234250	14:1802265	62.8	0.10	0.61
	rs109326954	14:1802266	62.8	0.10	0.61
*STAT5A*	rs109487069	19:43047829			
*GH*	**rs109191047**	19:48768766	97.5	0.19	0.19
	**rs137651874**	19:48769040	98.1	0.48	0.10
*FASN*	rs208645216	19:51400139			
	rs41919985	19:51402032	78.7	0.15	2.10^−3^
*GHR*	**rs385640152**	20:31909478	98.8	0.09	0.84
*PRLR*	**rs135164815**	20:39115344	99.2	0.15	0.91
*UTMP*	**rs132991801**	21:59667572	96.0	0.42	0.03
*LTF*	**rs41256920**	22:53521978	99.2	0.23	0.39
	**rs43706485**	22:53522038	95.5	0.19	0.93
*PRL*	**rs211032652**	23:35106206	99.5	0.08	0.57
	**rs110494133**	23:35110974	97.4	0.21	3.10^−6^
*SCD1*	**rs41255693**	26:21144708	98.9	0.19	0.17

^a^OPN3907 is a T deletion, not a SNP

Call rate, MAF and HWE *p*-value for the SNPs that did not present failure in the genotyping reactions are indicated. Markers bolded in black passed the quality control checks. Location of the SNPs is based on assembly UMD3.1

DNA sequencing of the sires allowed us to detect previously reported SNPs in bovine *LEP, ABCG2, OPN, PPARGC1A, CSN1S1, CSN2, CSN3, LGB, DGAT1, GH, GHR, PRLR, LTF,* and *PRL* genes. However, we did not identify any new SNP in these regions.

### Genotyping

Five out of the 39 markers included in the panel were discarded (OPN3907, rs41255680, rs43691049, rs109487069, and rs208645216) because of genotyping errors/failure in the genotyping reactions. For the remaining 34 SNPs, the overall success rate, calculated as the ratio between genotype calls and genotyped loci, was 0.94. However, 12 markers were removed because they failed to pass call rate (rs109326954, rs109234250, rs41255685, rs41919985, rs109625649, CSN1_AB, and rs109019599) or MAF (rs43702337, rs43706475, rs433385179, rs43703017, and rs43703013) thresholds. The SNPs rs43706475 and rs433385179 were monomorphic in the study population. Call rate, MAF, and HWE *p*-value for these 34 SNPs are indicated in Table 1. Additional file 2 shows the genotypic and allelic frequencies obtained for the 22 remaining SNPs, considered for subsequent analyses. Frequencies were discriminated for Holstein and Holstein x Jersey cows, without differentiating the degree in the cross.

An estimated 1.24% of the cows’ assigned genotypes showed Mendelian inconsistencies with their parent genotypes and consequently were considered as missing genotypes in the subsequent analysis. After filtering on call rate, 1346 out of 1618 cows were conserved for the association test.

### Prediction of 305-day milk production

When the whole population was evaluated, the phenotypic mean and the standard deviation for first lactation 305-day cumulative milk production predictions were 5967.38 and 880.52 kg, respectively. Furthermore, considering Holstein and Holstein x Jersey cows separately, phenotypic means and standard deviations were 6191.27 ± 806.76 and 5427.73 ± 815.05 kg, respectively.

### Statistical analysis and association test

When performing the association study on the whole population, every effect considered in the linear mixed model used was significant. Even though no significant associations after Bonferroni correction were detected in this test, 5 out of the 22 SNPs assessed showed suggestive associations with the 305-day cumulative milk production (Table 2). Those associations were detected on bovine autosomes 4, 6, 19, and 26. The greatest number of associations was found on BTA4, with two associations (rs29004488 and rs43375517), while the other three chromosomes (BTA6, 19, and 26) revealed a single SNP associated with the trait (rs132812135, rs137651874, and rs41255693, respectively). These five SNPs correspond to nucleotide variations occurring in *ARL4A, LEP, OPN, GH,* and *SCD1* genes.

**Table 2:** SNP markers associated with milk production

SNP	BONF/BH adjusted *p*-values			Gene	Variation
	WP	HP	HxJ P		
**rs43375517**	0.06/0.06	0.01*/0.01	1/0.86	ARL4A	C/G, 5’UTR
**rs41255693**	0.33/0.09	0.43/0.09	1/0.87	SCD1	C/T, missense
**rs29004488**	0.40/0.09	1/0.50	0.27/0.14	LEP	T/C, missense
**rs137651874**	0.46/0.09	0.52/0.09	1/0.77	GH	G/A, intronic
**rs132812135**	0.47/0.09	1/0.20	1/0.66	OPN	A/C, 3’UTR
**rs135164815**	1/0.21	0.03*/0.02	0.98/0.20	PRLR	G/A, missense
rs43703011	1/0.21	1/0.26	0.94/0.20	CSN2	G/T, missense
**rs43706485**	1/0.22	0.07/0.02	1/0.30	LTF	C/G, 5’UTR
**rs109191047**	1/0.22	0.56/0.09	1/0.67	GH	A/C, synonymous
rs110930453	1/0.35	1/0.54	1/0.77	OPN	C/T, intronic
rs385640152	1/0.35	1/0.35	1/0.86	GHR	A/T, missense
rs110494133	1/0.35	1/0.60	1/0.53	PRL	C/T, intronic
rs132991801	1/0.36	1/0.60	1/0.77	UTMP	C/T, synonymous
rs211032652	1/0.36	1/0.26	1/0.86	PRL	G/A, synonymous
rs41595314	1/0.48	1/0.50	1/0.86	SNX13	A/T, intronic
rs42213673	1/0.53	1/0.60	1/0.86	ETV1	G/A, intronic
rs17870811	1/0.53	1/0.17	0.24/0.14	PPARGC1A	C/T, intronic
rs109579682	1/0.53	1/0.95	0.78/0.20	PPARGC1A	A/G, intronic
rs133669403	1/0.53	1/0.60	1/0.77	PPARGC1A	G/A, missense
rs43703015	1/0.53	1/0.34	1/0.77	CSN3	C/T, missense
rs41256920	1/0.86	1/0.60	1/0.67	LTF	C/A, 5’UTR
rs43703010	1/0.90	1/0.60	1/0.77	CSN1S1	A/G, missense

*p < 0.05

This Table indicates markers adjusted *p*-values based on Bonferroni correction (BONF) and Benjamini-Hochberg procedure (BH) for the whole (WP), Holstein (HP) and Holstein x Jersey (HxJ P) population analyses, corresponding gene names, and nucleotide changes implicated in the polymorphisms. SNPs in bold represent suggestive or significant associations with 305-day milk production

Regarding the Holstein subpopulation, the SNPs rs43375517, located in *ARL4A* (BTA4), and rs135164815, located in *PRLR* (BTA20), were significantly associated with 305-day milk production after Bonferroni correction. Furthermore, other four SNPs showed suggestive associations. Two of them, rs137651874 and rs41255693, were also detected when analyzing the total population, while rs109191047 and rs43706485 were only detected when considering the Holstein population for analysis. SNP rs109191047 is a synonymous substitution in *GH* gene and rs43706485 is a nucleotide variation in the 5′ untranslated region of *LTF* gene. This association test was performed using a linear model considering the fixed effects of breed, year of birth, sire, and the combined effect of season and year of first lactation. The fixed herd effect did not show to be significant in this analysis. When performing the association study on the Holstein x Jersey crosses subpopulation, the combined effect of season and year of first lactation was not found to be significant, so only the effects of the covariates breed, year of birth, sire and herd were considered in the model used. In this test, none SNP showed a Benjamini-Hochberg adjusted *p*-value smaller than the false discovery rate used.

**Fig. 1:**
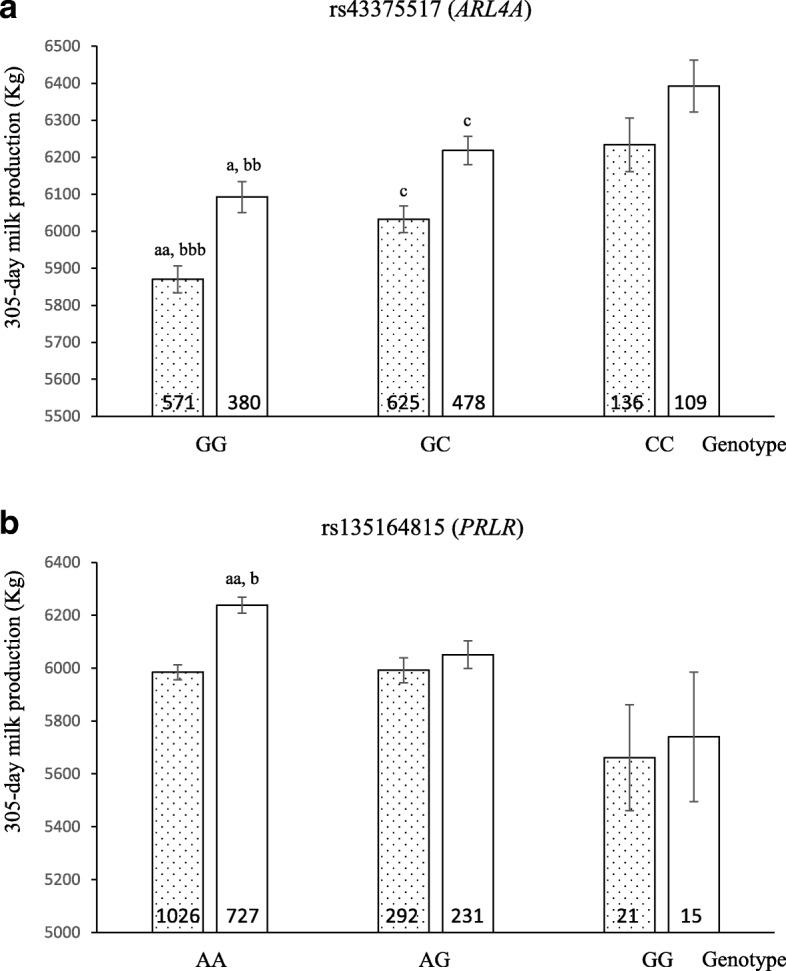
Effect of different SNP genotypes on milk production. Genotypes for SNPs from *ARL4A* (**a**) and *PRLR* (**b**) genes are shown in the *x*-axis and 305-day cumulative milk production (Kg) in the *y*-axis. Dot bars and white bars denote the whole population and the Holstein subpopulation, respectively. The number of animals presenting each genotype is indicated inside each bar. Homozygote GG for rs43375517 or AA for rs135164815 vs heterozygote, ^aa^*p* < 0.005; ^a^*p* < 0.05. Homozygote 1 vs homozygote 2, ^bbb^*p* < 0.0005; ^bb^*p* < 0.005; ^b^*p* < 0.05. Heterozygote vs homozygote CC for rs43375517 or GG for rs135164815, ^c^*p* < 0.05

To illustrate the effect of each significantly associated SNP genotype on milk production, we compared 305-day cumulative milk production values of the cows with each of the three possible genotypes. As shown in Fig. 1, the cows with genotype GG in SNP rs43375517 (*ARL4A*) presented lower cumulative milk production than those with the other genotypes (*p* < 0.05 and *p* < 0.005 when comparing homozygous GG vs. heterozygous, and vs. homozygous CC, respectively). Regarding rs135164815 (*PRLR*), Holstein cows with genotype AA presented higher cumulative milk production than those with the other genotypes (*p* < 0.005 and *p* < 0.05 when comparing homozygous AA vs. heterozygous, and vs. homozygous GG, respectively). We further determined the population frequencies for the favorable alleles of these two SNPs found to be significantly associated with milk production. They were 0.34 for allele C of SNP rs43375517, and 0.88 for allele A of SNP rs135164815.

## Discussion

The economic relevance of lactation and milk production has given rise to numerous research studies on polymorphisms in candidate genes associated with these traits. In dairy cattle, much research has focused on the association of certain genetic variants of milk proteins with milk yield and composition traits [42]. Major milk protein genes have been identified in different genetic variants encoding similar proteins that are slightly different in chemical structure [38]. Based on this information, we selected 23 candidate genes identified in numerous independent studies that used the same or different approaches. Furthermore, we selected 46 SNPs among these candidate genes for the SNPlex genotyping panel design.

The quality control test performed considerably reduced the number of candidate SNPs, mainly because of a low call rate and failure in the genotyping reactions. However, the proportion of discarded SNPs was similar to that of previously reported studies which used the same genotyping system [43–47], even though some of them [43–45] set a less stringent minimum call rate than the one used in this study. Herein, SNPs rs109234250 and rs109326954, located in the *DGAT* gene, showed the highest percentage of missing genotypes (37.2%) and therefore we did not take them into account for further analysis, although *DGAT* is a major QTL for milk production traits. Likewise, we could not evaluate the association of *LGB* variants with milk yield as we removed the four SNPs within this candidate gene locus due to the genotyping errors in the reactions (rs41255680 and rs43691049) or low call rate (rs41255685 and rs109625649) that we found.

Based on the genotypes that passed the quality control criteria, the association test performed on the total population revealed that 22.7% of the SNPs evaluated (5 out of 22 SNPs) showed suggestive associations (adjusted Benjamini-Hochberg *p*-values < 0.1) with milk production. This high percentage was not surprising because the markers analyzed were located at candidate genes or regions, or they had been previously reported to be associated with bovine milk production or composition traits. To provide additional information and to detect associations which may only occur in either the Holstein or the Holstein x Jersey subpopulations, we performed separate association analyses discriminating animals by breed. Regarding Holstein x Jersey animals, no significant SNPs were identified after multiple testing correction. However, when testing the Holstein subpopulation, two SNPs, rs43375517 and rs135164815, reached significance. The former is located in the 5’untranslated region (5’UTR) of *ARL4A* gene. This SNP was previously associated with predicted transmitting ability for milk in Holstein bulls [32]. The latter SNP constitutes a missense mutation in *PRLR* gene, which was previously reported by Viitala et al. as influencing milk protein and fat yield [33]. We found that the individuals with better performance were those with CC genotype at locus rs43375517 and AA genotype at locus rs135164815. Furthermore, when performing the association test on the Holstein population, other four SNPs showed suggestive associations to milk production according to the Benjamini-Hochberg false discovery rate test, These markers were rs43706485 in *LTF* gene, rs137651874 and rs109191047 both located in *GH* gene, and rs41255693 in *SCD1* gene.

Three of the SNPs identified (rs135164815, rs41255693, and rs29004488) correspond to non-synonymous mutations in *PRLR*, *SCD1*, and *LEP* genes. These missense variations could directly affect the protein biological function. Another suggestive SNP, rs137651874, is an intronic variation of *GH* gene. This polymorphism could trigger an abnormal splicing of mRNA, i.e. making a normal splice site disappear or generating an alternative one, or it could be transcribed to small regulatory RNAs, like micro RNAs, which can regulate gene expression. The SNP rs109191047, a synonymous mutation of *GH* gene, although it does not alter the primary sequence of the protein, it could generate a cryptic or alternative splice site of mRNA, could affect the translation rate based on tRNA availability for the new codon, or could simply be in linkage disequilibrium with another SNP that actually affects the phenotype. The remaining SNPs detected, rs43375517, rs132812135, and rs43706485 are located in the 5’UTR of *ARL4A,* in the 3’UTR of *OPN* and in the 5’UTR of *LTF*, respectively, and could be affecting post-transcriptional regulation of gene expression.

There is abundant reported evidence that *OPN, LEP, ARL4A, GH, PRLR,* and *SCD1* genes are associated with milk production and/or composition traits [26, 30, 32, 33, 41, 48, 49]. Therefore, the results obtained in this study confirmed previously reported associations in other populations. Moreover, these results evidence the so-called “holsteinization” process, which has taken place in Latin America since the 1980s. In Argentina in particular, 65% of dairy cows are inseminated with Holstein semen imported from the United States and Canada. Consequently, it is not surprising that the associations found herein have been previously reported by other authors, based on the common genetic background being due to importations not only of semen but also of embryos and live animals from the northern hemisphere [50].

The approach followed in this study is a plausible way to explore SNPs affecting specific traits, such as milk production. Thereafter, these SNPs already proven to have a significant effect on the desired trait could be suitable as possible candidates for marker-assisted selection (MAS) programs. Moreover, they could even help to develop low density/low cost customized genotyping panels containing a few hundred or a few thousand markers, to be routinely used assisting breeding programs. In the context in which this work was started and developed, it was more cost-effective to genotype a few SNPs on candidate genes than to perform large-scale genotyping. However, with technological advancement in terms of development, availability and cost reduction of high-throughput microarray genotyping, Genomic Selection (GS) has become more affordable. In fact, in several countries, it is customary to use GS based on low or medium density genotyping in dairy cattle breeding. According to genome-wide association studies and genomic selection estimations, dairy traits are affected by 2000–10,000 genes, with the concomitant implication that if many genes affect a trait, individual genes have small effects, thus limiting the efficiency of the MAS approach [51]. Hence, whenever possible, Genomic Selection should be used since it captures a greater amount of the phenotypic variance. However, in a context where economic resources are scarce, MAS would be still applicable.

## Conclusions

This is the first study reporting associations between genetic markers and milk production in Argentinean Holstein and Holstein x Jersey cows. It not only provides useful information to explore the most relevant genes contributing to the variation in bovine milk production, but also, and most important at country level, it might constitute a first step towards the design of selection programs aimed at increasing profitability of dairy operations by improving milk production performance of Argentinean Holstein and Holstein x Jersey dairy cattle.

## Additional file

Additional file 1: Primer sets for PCR used for amplification of 14 candidate genes on sire DNA. (DOC 109 kb)

Additional file 2: Genotypic and allelic frequencies obtained for the SNPs that passed the quality control. (DOC 62 kb)

## Abbreviations

BTA: *Bos taurus* autosome
GS: Genomic Selection
HWE: Hardy-Weinberg equilibrium
MAF: Minor allele frequency
MAS: Marker-assisted selection
QTL: Quantitative trait loci
SNP: Single nucleotide polymorphism
