# Supplementary material for: Single nucleotide polymorphisms in candidate genes associated with milk yield in Argentinean Holstein and Holstein x Jersey cows
Source: J Anim Sci Technol. 2018 Dec 12;60:31. doi: 10.1186/s40781-018-0189-1 (PMC6291960; doi:10.1186/s40781-018-0189-1)
Supplement: Supplementary file 1 — Primer sets for PCR used for amplification of 14 candidate genes on sire DNA. (DOC 109 kb) [file 40781_2018_189_MOESM1_ESM.doc]

**Single nucleotide polymorphisms in candidate genes associated with milk yield in Argentinean Holstein and Holstein x Jersey cows**

**María A Raschia^1*^, Juan P Nani^2^, Daniel O Maizon^3^, María J Beribe^4^, Ariel F Amadio^2,5^, Mario A Poli^1^.**

1 Instituto de Genética “Ewald A. Favret”, CICVyA-CNIA, Instituto Nacional de Tecnología Agropecuaria, Nicolás Repetto y de Los Reseros s/n, Hurlingham (B1686), Buenos Aires, Argentina.

2 E.E.A. Rafaela, Instituto Nacional de Tecnología Agropecuaria, Ruta 34 Km 227, Rafaela, Santa Fe, Argentina.

3 E.E.A. Anguil, Instituto Nacional de Tecnología Agropecuaria, Ruta 5 Km 580, Anguil, La Pampa, Argentina.

4 E.E.A. Pergamino, Instituto Nacional de Tecnología Agropecuaria, Ruta 32 Km 4.5, Pergamino, Buenos Aires, Argentina.

5 Consejo Nacional de Investigaciones Científicas y Técnicas, Argentina.

* e-mail: raschia.maria@inta.gob.ar

**Additional file 1** Primer sets for PCR used for amplification of 14 candidate genes on sire DNA

| **Gene** | **GenBank accession number** | **Primers sequence** | **AT** | **Amplicon (bp)** |
| --- | --- | --- | --- | --- |
| *PRLR* | NW_001493958 | F 5’-TACTGGAGTGGGTTGCCATT-3’ | 60 | 353 |
|  |  | R 5’-TTCTTCACCTGTCACGCTTC-3’ | 59 |  |
|  |  | F 5’-GTGCTCCATGGCAAGAGAAG-3’ | 61 | 446 |
|  |  | R 5’-CACTAACCCCAACCTTCCAC-3’ | 59 |  |
|  |  | F 5’-TCAGCAAGGAGCAAGAATCA-3’ | 60 | 452 |
|  |  | R 5’-TGGAAGTCAGAGCATGGTGA-3’ | 60 |  |
| *PRL* | AF426315 | F 5’-TTTGCAGAACACAGGAGCAC-3’ | 60 | 489 |
|  |  | R 5’-ACGCAAAGGTTTCTTCTGGA-3’ | 60 |  |
| *GH* | NC_007317 | F 5’-CAACAGATGGCTGGCAACTA-3’ | 60 | 436 |
|  |  | R 5’-GCTTAGCCAGGAGAATGCAC-3’ | 60 |  |
| *LTF* | NC_007320 | F 5’-GTCTCCACCCCCACTCTTC-3’ | 59 | 419 |
|  |  | R 5’-CGTGTCAAACTGCCTGCTG-3’ | 62 |  |
| *DGAT1* | AJ318490 | F 5’-GCACCATCCTCTTCCTCAAG-3’ | 57 | 411 |
|  |  | R 5’-GGAAGCGCTTTCGGATG-3’ | 56 |  |
| *GHR* | NC_007318 | F 5’-GTGGCTATCAAGTGAAATCATTGAC-3’ | 60 | 342 |
|  |  | R 5’-ACTGGGTTGATGAAACACTTCACTC-3’ | 60 |  |
| *LEP* | NW_001494939 | F 5’-ACATCCGTTGTTCACTGTGGC-3’ | 61 | 3620 |
|  |  | R 5’-TGGTCCTTCAAGATCCATTCA-3’ | 60 |  |
|  |  | F 5’-AGGCTGTGCAGCCTTGCA-3’ | 64 | a |
|  |  | R 5’-AGGATTCCGGTCTGGGAG-3’ | 58 |  |
|  |  | F 5’-TGTGTGCCCTCTTTCAAGGT-3’ | 58 | a |
|  |  | R 5’-CTAACTGCTCTCTGGCTTCC-3’ | 58 |  |
|  |  | F 5’-CGATCTACCAACAGATCC-3’ | 55 | a |
|  |  | R 5’-CAAGCTCTCCAAGCTCTC-3’ | 55 |  |
| *ABCG2* | AJ871176 | F 5’-TCACGAGACTGTCAGGGACTT-3’ | 60 | 369 |
|  |  | R 5’-CAATCCTTCAGCTCCCTCAG-3’ | 60 |  |
| *OPN* | AY878328 | F 5’-GCTACCCCTAACTTCTGTTCCA-3’ | 59 | 424 |
|  |  | R 5’-TCGTGGGGTTTTTGAGAGAT-3’ | 59 |  |
|  |  | F 5’-GCCACAGAGGAGGACTTCAC-3’ | 60 | 764 |
|  |  | R 5’-CACAACTGATAGCACCAGACTCA-3’ | 60 |  |
| *PPARGC1A* | NW_001495167 | F 5’-GGATTCCCGCTTCTCATACTC-3’ | 60 | 414 |
|  |  | R 5’-TCCAGGGGCTACTCAGTCAT-3’ | 60 |  |
| *CSN3* | AY380228 | F 5’-TAGGTCACCTGCCCAAATTC-3’ | 60 | 462 |
|  |  | R 5’-ATTAGCCCATTTCGCCTTCT-3’ | 60 |  |
| *LGB* | X14710 | F 5’-GCAGAGCCCTCGATACTGAC-3’ | 60 | 615 |
|  |  | R 5’-CATGGTCTGGGTGACAATGA-3’ | 60 |  |
|  |  | F 5’-GGGTCAGAGTGCAGGAGAGA-3’ | 60 | 402 |
|  |  | R 5’-CACCATCGATCTTGAACACC-3’ | 59 |  |
|  |  | F 5’-GAAGGGGCCACACCATTTT-3’ | 62 | 435 |
|  |  | R 5’-CCGGTATATGACCACCCTCT-3’ | 59 |  |
|  |  | F 5’-GGGGACTTGGTACTCCTTGG-3’ | 60 | 1076 |
|  |  | R 5’-CACCATCGATCTTGAACACC-3’ | 59 |  |
|  |  | F 5’-GAAGGGGCCACACCATTTT-3’ | 62 | 1374 |
|  |  | R 5’-CCCTACCCCATGTGTGTGC-3’ | 62 |  |
| *CSN2* | NC_007304 | F 5’-TGAGTTGACTGTGGGAACTAAAG-3’ | 58 | 400 |
|  |  | R 5’-GTGAAAGGGAAGTCGCTCAG-3’ | 60 |  |
|  |  | F 5’-CAGCCTTATTCAGAAGAGTGGAA-3’ | 59 | 450 |
|  |  | R 5’-TCAAAGTTTTTATTTCTTGCACTGA-3’ | 60 |  |
|  |  | F 5’-TGGATTATGGACTCAAAGATTTGTT-3’ | 60 | 500 |
|  |  | R 5’-GGCTCCTGGTACAGCAGAAA-3’ | 60 |  |
| *CSN1S1* | X59856 | F 5’-GGCCTGGTAAAGAAGGAACC-3’ | 60 | 469 |
|  |  | R 5’-TGCACAAAACAGCGTAAGTG-3’ | 59 |  |
|  |  | F 5’-AGTTAGCCTGGTAGGTAGGCT-3’ | 56 | 1820 |
|  |  | R 5’-TGCCTTATGCAATGAAACCTT-3’ | 60 |  |
|  |  | F 5’-TGTGATGCGAATAGCCATGT-3’ | 60 | 460 |
|  |  | R 5’-CACTGCTCCACATGTTCCTG-3’ | 60 |  |

^a^ PCR walking primers used only for sequencing purposes

AT: annealing temperature
