# Supplementary material for: Single nucleotide polymorphisms in candidate genes associated with milk yield in Argentinean Holstein and Holstein x Jersey cows
Source: J Anim Sci Technol. 2018 Dec 12;60:31. doi: 10.1186/s40781-018-0189-1 (PMC6291960; doi:10.1186/s40781-018-0189-1)
Supplement: Supplementary file 2 — Genotypic and allelic frequencies obtained for the SNPs that passed the quality control. (DOC 62 kb) [file 40781_2018_189_MOESM2_ESM.doc]

**Single nucleotide polymorphisms in candidate genes associated with milk yield in Argentinean Holstein and Holstein x Jersey cows**

**María A Raschia^1*^, Juan P Nani^2^, Daniel O Maizon^3^, María J Beribe^4^, Ariel F Amadio^2,5^, Mario A Poli^1^.**

1 Instituto de Genética “Ewald A. Favret”, CICVyA-CNIA, Instituto Nacional de Tecnología Agropecuaria, Nicolás Repetto y de Los Reseros s/n, Hurlingham (B1686), Buenos Aires, Argentina.

2 E.E.A. Rafaela, Instituto Nacional de Tecnología Agropecuaria, Ruta 34 Km 227, Rafaela, Santa Fe, Argentina.

3 E.E.A. Anguil, Instituto Nacional de Tecnología Agropecuaria, Ruta 5 Km 580, Anguil, La Pampa, Argentina.

4 E.E.A. Pergamino, Instituto Nacional de Tecnología Agropecuaria, Ruta 32 Km 4.5, Pergamino, Buenos Aires, Argentina.

5 Consejo Nacional de Investigaciones Científicas y Técnicas, Argentina.

* e-mail: raschia.maria@inta.gob.ar

**Additional file 2** Genotypic and allelic frequencies obtained for the SNPs that passed the quality control

| **SNP** | **Holstein** | | | | | **Holstein x Jersey crosses** | | | | |
| --- | --- | --- | --- | --- | --- | --- | --- | --- | --- | --- |
|  | **Genotypic frequency** | | | **Allelic Frequency** | | **Genotypic frequency** | | | **Allelic frequency** | |
|  | **AA** | **AB** | **BB** | **A** | **B** | **AA** | **AB** | **BB** | **A** | **B** |
| rs43375517 | 0.11 | 0.49 | 0.40 | 0.36 | 0.64 | 0.07 | 0.40 | 0.52 | 0.28 | 0.72 |
| rs42213673 | 0.15 | 0.48 | 0.36 | 0.40 | 0.60 | 0.05 | 0.41 | 0.53 | 0.26 | 0.74 |
| rs41595314 | 0.00 | 0.12 | 0.88 | 0.06 | 0.94 | 0.00 | 0.10 | 0.90 | 0.05 | 0.95 |
| rs29004488 | 0.16 | 0.51 | 0.33 | 0.41 | 0.59 | 0.22 | 0.55 | 0.23 | 0.49 | 0.51 |
| rs132812135 | 0.10 | 0.44 | 0.47 | 0.31 | 0.69 | 0.02 | 0.33 | 0.65 | 0.18 | 0.82 |
| rs110930453 | 0.25 | 0.50 | 0.25 | 0.50 | 0.50 | 0.14 | 0.48 | 0.39 | 0.38 | 0.63 |
| rs109579682 | 0.03 | 0.36 | 0.61 | 0.21 | 0.79 | 0.04 | 0.34 | 0.63 | 0.21 | 0.79 |
| rs133669403 | 0.00 | 0.12 | 0.88 | 0.06 | 0.94 | 0.04 | 0.58 | 0.38 | 0.33 | 0.67 |
| rs17870811 | 0.00 | 0.16 | 0.83 | 0.09 | 0.91 | 0.01 | 0.09 | 0.90 | 0.05 | 0.95 |
| rs43703010 | 0.00 | 0.05 | 0.95 | 0.03 | 0.97 | 0.04 | 0.44 | 0.52 | 0.26 | 0.74 |
| rs43703011 | 0.12 | 0.50 | 0.38 | 0.37 | 0.63 | 0.09 | 0.45 | 0.46 | 0.32 | 0.68 |
| rs43703015 | 0.00 | 0.17 | 0.83 | 0.09 | 0.91 | 0.05 | 0.44 | 0.51 | 0.27 | 0.73 |
| rs109191047 | 0.02 | 0.25 | 0.73 | 0.15 | 0.85 | 0.01 | 0.12 | 0.87 | 0.07 | 0.93 |
| rs137651874 | 0.19 | 0.51 | 0.30 | 0.44 | 0.56 | 0.08 | 0.56 | 0.35 | 0.37 | 0.63 |
| rs385640152 | 0.03 | 0.28 | 0.70 | 0.16 | 0.84 | 0.01 | 0.19 | 0.80 | 0.10 | 0.90 |
| rs135164815 | 0.01 | 0.23 | 0.75 | 0.13 | 0.87 | 0.02 | 0.17 | 0.82 | 0.10 | 0.90 |
| rs132991801 | 0.06 | 0.43 | 0.50 | 0.28 | 0.72 | 0.14 | 0.61 | 0.26 | 0.44 | 0.56 |
| rs41256920 | 0.01 | 0.25 | 0.74 | 0.14 | 0.86 | 0.13 | 0.56 | 0.31 | 0.41 | 0.59 |
| rs43706485 | 0.02 | 0.26 | 0.73 | 0.14 | 0.86 | 0.11 | 0.48 | 0.42 | 0.35 | 0.65 |
| rs211032652 | 0.01 | 0.16 | 0.83 | 0.09 | 0.91 | 0.02 | 0.26 | 0.73 | 0.15 | 0.85 |
| rs110494133 | 0.06 | 0.44 | 0.49 | 0.28 | 0.72 | 0.05 | 0.49 | 0.46 | 0.29 | 0.71 |
| rs41255693 | 0.02 | 0.28 | 0.70 | 0.16 | 0.84 | 0.00 | 0.18 | 0.82 | 0.09 | 0.91 |

Frequencies were discriminated for pure Holstein cows and Holstein x Jersey crosses. Animals and SNPs with more than 10% missing genotypes were discarded. SNPs with MAF below 0.05 were also excluded.
